# Supplementary material for: Restoration Efficacy of Picea likiangensis var. rubescens Rehder & E. H. Wilson Plantations on the Soil Microbial Community Structure and Function in a Subalpine Area
Source: Microorganisms. 2021 May 26;9(6):1145. doi: 10.3390/microorganisms9061145 (PMC8226860; doi:10.3390/microorganisms9061145)
Supplement: Supplementary file 1 [file microorganisms-09-01145-s001.zip › microorganisms-1185406-supplementary.pdf]

Table S1. Relative abundance (%) of individual order of the bacterial communities in the three stands

Order	25-year-old	40-year-old	PCF
Acidobacteriales	14.36 (±1.75)a	23.45 (±3.40)a	22.91 (±1.49)a
Rhizobiales	**21.07 (±0.76)a**	**20.13 (±0.55)a**	**16.44 (±0.84)b**
Burkholderiales	8.24 (±0.48)a	8.73 (±0.55)a	8.57 (±2.07)a
Rhodospirillales	4.19 (±0.21)a	4.44 (±0.71)a	4.42 (±0.36)a
Solibacterales	2.73 (±0.30)a	2.45 (±0.42)a	4.19 (±1.07)a
Xanthomonadales	2.29 (±0.02)a	1.63 (±0.06)b	3.99 (±2.10)ab
Sphingomonadales	2.16 (±0.11)a	3.42 (±0.94)a	2.09 (±0.25)a
Caulobacterales	1.78 (±0.14)a	2.91 (±0.79)a	2.88 (±0.42)a
Sphingobacteriales	2.86 (±0.37)a	2.52 (±0.98)a	1.78 (±0.40)a
Corynebacteriales	2.74 (±0.18)a	1.71 (±0.44)a	2.10 (±0.36)a
Myxococcales	1.47 (±0.09)a	1.54 (±0.39)a	2.28 (±0.77)a
Unclassified_d__Bacteria	1.96 (±0.04)a	1.44 (±0.41)a	1.58 (±0.29)a
Streptomycetales	1.89(±0.16)a	1.21 (±0.32)a	1.19 (±0.07)a
Unclassified_c__Spartobacteria	1.59 (±0.13)a	1.18 (±0.59)a	1.19 (±0.20)a
Unclassified_p__Acidobacteria	1.67 (±0.11)a	1.10 (±0.31)a	1.05 (±0.26)a
Streptosporangiales	1.54 (±0.15)a	0.93 (±0.29)a	0.87 (±0.09)a
Verrucomicrobiales	0.87 (±0.07)a	0.98 (±0.29)a	1.34 (±0.24)a
Nevskiales	0.76 (±0.05)a	1.13 (±0.29)a	1.10 (±0.12)a
Solirubrobacterales	**1.72 (±0.27)a**	**0.61 (±0.20)b**	**0.51 (±0.06)b**
Unclassified_c__Gammaproteobacteria	0.81 (±0.05)a	1.00 (±0.23)a	0.98 (±0.13)a
Unclassified_c__Betaproteobacteria	1.15 (±0.06)a	0.66 (±0.18)a	0.56 (±0.19)a
Unclassified_c__Blastocatellia	0.97 (±0.13)a	0.86 (±0.50)a	0.55 (±0.14)a
Gemmatimonadales	0.99 (±0.02)a	0.81 (±0.31)a	0.52 (±0.12)a
Planctomycetales	0.72 (±0.05)a	0.63 (±0.14)a	0.96 (±0.20)a
Pseudonocardiales	**0.98 (±0.09)a**	**0.57 (±0.15)ab**	**0.53 (±0.02)b**
Pseudomonadales	0.53 (±0.02)a	0.66 (±0.05)a	0.78 (±0.13)a
Micrococcales	**0.95 (±0.10)a**	**0.43 (±0.08)b**	**0.40 (±0.02)b**
Flavobacteriales	0.68 (±0.18)a	0.30 (±0.04)a	0.26 (±0.02)a

Data are presented as the mean value with the standard error (SE) given in parenthesis (n=3). Mean values within a row followed by different lowercase letters are significantly different at $p < 0.05$. 25-year-old, 40-year-old, and PCF represent 25-year-old *P. rubescens* plantation, 40-year-old *P. rubescens* plantation, and primeval coniferous forest, respectively. Data analysis was based on one-way analysis of variance (ANOVA) with Tukey's test ($p<0.05$).

Table S2. Relative abundance (%) of individual order of the fungal communities in the three stands

Order	25-year	40-year	PCF
Agaricales	18.19 (±2.07)a	43.94 (±17.06)a	21.07 (±0.46)a
Russulales	**3.63 (±0.32)a**	**1.71 (±0.50)a**	**31.99 (±4.46)b**
Polyporales	**6.30 (±0.84)a**	**4.90 (±1.11)a**	**13.97 (±1.22)b**
Boletales	6.42 (±0.49)a	4.69 (±1.20)a	7.96 (±0.39)a
Atheliales	6.99 (±1.18)a	5.88 (±1.93)a	5.76 (±2.00)a
Helotiales	7.31 (±2.96)a	15.57 (±8.77)a	1.40 (±1.07)a
Unclassified_c__Leotiomycetes	4.43 (±1.71)a	4.74 (±2.49)a	1.10 (±0.65)a
Hypocreales	3.33 (±0.74)a	2.46 (±0.95)a	1.74 (±0.48)a
Gloeophyllales	**0.81 (±0.10)a**	**0.54 (±0.17)a**	**2.32 (±0.32)b**
Cantharellales	4.37 (±1.03)a	0.91 (±0.20)a	1.69 (±0.03)a
Eurotiales	4.39 (±1.02)a	2.34 (±0.98)a	0.80 (±0.24)a
Jaapiales	0.60 (±0.16)a	0.71 (±0.37)a	1.53 (±0.21)a
Corticiales	**0.87 (±0.24)a**	**0.40 (±0.01)b**	**1.30 (±0.16)a**
Chaetothyriales	3.06 (±0.72)a	1.05 (±0.62)a	0.68 (±0.14)a
Glomerales	2.96 (±0.83)a	0.87 (±0.42)a	0.62 (±0.50)a
Geastrales	**1.41 (±0.42)a**	**0.55 (±0.07)b**	**0.63 (±0.03)a**
Sebacinales	**5.99 (±4.97)a**	**0.14 (±0.02)b**	**0.22 (±0.04)b**
Sordariales	1.26 (±0.31)a	0.80 (±0.12)a	0.41 (±0.07)a
Erysiphales	0.35 (±0.24)a	1.34 (±0.66)a	0.19 (±0.18)a
Pleosporales	1.56 (±0.41)a	0.81 (±0.35)a	0.19 (±0.07)a
Hymenochaetales	0.70 (±0.44)a	0.07 (±0.03)a	0.53 (±0.06)a
Mucorales	**1.92 (±0.41)a**	**0.39 (±0.16)b**	**0.34 (±0.18)b**
Glomerellales	**1.45 (±0.23)a**	**0.60 (±0.24)b**	**0.22 (±0.02)b**
Auriculariales	0.59 (±0.26)a	0.35 (±0.20)a	0.36 (±0.00)a
Onygenales	0.95 (±0.21)a	0.29 (±0.12)a	0.32 (±0.12)a
Botryosphaeriales	0.35 (±0.12)a	0.60 (±0.30)a	0.08 (±0.03)a
Xylariales	0.83 (±0.15)a	0.16 (±0.05)a	0.10 (±0.03)a
Monoblepharidales	1.14 (±0.25)a	0.14 (±0.05)a	0.08 (±0.02)a
Pucciniales	0.70 (±0.26)a	0.10 (±0.06)a	0.10 (±0.01)a

Data are presented as the mean value with the standard error (SE) given in parenthesis (n=3). Mean values within a row followed by different lowercase letters are significantly different at $p < 0.05$. 25-year-old, 40-year-old, and PCF represent 25-year-old *P. rubescens* plantation, 40-year-old *P. rubescens* plantation, and primeval coniferous forest, respectively. Data analysis was based on one-way analysis of variance (ANOVA) with Tukey's test ($p<0.05$).

Table S3. Relative abundance (%) of individual order of the archaeal communities in the three stands

Order	25-year	40-year	PCF
Methanosarcinales	21.16 (±0.84)a	23.96 (±1.08)a	22.32(±1.08)a
Methanomicrobiales	7.00 (±0.08)a	8.86 (±0.70)ab	8.48 (±0.11)b
Nitrososphaerales	**6.07 (±0.22)a**	**8.21 (±0.34)b**	**6.32 (±0.32)a**
Unclassified_d__Archaea	5.96 (±0.24)a	5.95 (±0.37)a	6.79 (±0.42)a
Halobacteriales	**7.16 (±0.19)a**	**5.44 (±0.22)b**	**5.17 (±0.37)b**
Unclassified_p__Euryarchaeota	4.72 (±0.22)a	4.44 (±0.28)a	4.79 (±0.04)a
Unclassified_p__Candidatus_Bathyarchaeota	**5.04 (±0.22)a**	**4.16 (±0.06)b**	**4.79 (±0.06)a**
Methanobacteriales	4.45 (±0.08)a	4.46 (±0.10)a	4.46 (±0.34)a
Natrialbales	4.78 (±0.32)a	5.07 (±0.77)a	3.87 (±0.41)a
Thermoplasmatales	4.40 (±0.17)a	4.03 (±0.48)a	3.62 (±0.05)a
Unclassified_p__Thaumarchaeota	4.77 (±0.04)a	3.72 (±0.30)a	3.46 (±0.23)a
Haloferacales	**4.82 (±0.38)a**	**3.90 (±0.25)ab**	**3.14 (±0.35)b**
Methanocellales	3.57 (±0.08)a	3.07 (±0.17)a	3.31 (±0.22)a
Thermococcales	3.15 (±0.21)a	2.56 (±0.26)a	3.02 (±0.29)a
Thermoproteales	**1.78 (±0.14)a**	**1.20 (±1.16)a**	**2.68 (±0.22)b**
Sulfolobales	**0.74 (±0.04)a**	**0.97 (±0.13)a**	**3.23 (±0.29)b**
Archaeoglobales	1.39 (±0.09)a	1.42 (±0.10)a	1.75 (±0.14)a
Unclassified_p__Candidatus_Thorarchaeota	1.23 (±0.08)a	1.44 (±0.06)a	1.16 (±0.10)a
Nitrosopumilales	1.29 (±0.07)a	1.27 (±0.24)a	1.10 (±0.11)a
Unclassified_c__Hadesarchaea	1.12 (±0.07)a	0.96 (±0.14)a	1.31 (±0.19)a
Methanococcales	1.07 (±0.24)a	0.88 (±0.09)a	1.04 (±0.09)a
Methanomassiliicoccales	**0.97 (±0.12)ab**	**0.71 (±0.02)a**	**0.90 (±0.03)b**
Unclassified_p__Candidatus_Lokiarchaeota	0.76 (±0.05)a	0.77 (±0.07)a	0.87 (±0.15)a
Unclassified_c__Methanomicrobia	0.53 (±0.16)a	0.94 (±0.09)a	0.61 (±0.01)a
unclassified_p__Candidatus_Micrarchaeota	0.12 (±0.04)a	0.09 (±0.01)a	0.48 (±0.37)a

Data are presented as the mean value with the standard error (SE) given in parenthesis (n=3). Mean values within a row followed by different lowercase letters are significantly different at $p < 0.05$. 25-year-old, 40-year-old, and PCF represent 25-year-old *P. rubescens* plantation, 40-year-old *P. rubescens* plantation, and primeval coniferous forest, respectively. Data analysis was based on one-way analysis of variance (ANOVA) with Tukey's test (p<0.05).
